# Pushing the boundaries of robotic computed tomography: automated twin-robot CT scan with maximum reachability

**DOI:** 10.1038/s41598-026-56718-9

**Published:** 2026-07-02

**Authors:** Jonas Schnitzer, Vinayaka Raju Sathyanarayana Raju, Nihat Emir Erdebil, Gabriel Herl, Andreas Maier

**Affiliations:** 1 Friedrich-Alexander-University, Pattern Recognition Lab, Erlangen, 91058 Martenstraße 3, Bayern Germany; 2 BMW Group, Technology Material and Process Analytics, München, Petuelring 130, 80809 Germany; 3 TU Dortmund, Electrical Engineering and Information Technology, Otto-Hahn-Straße 4, 44227 Dortmund, Germany; 4 TU München, School of Computation, Information and Technology, Arcisstraße 21, 80333 München, Germany; 5 Deggendorf Institute of Technology, Deggendorf, 94469 Dieter-Görlitz Platz 1, Germany

**Keywords:** Robotic CT systems, Trajectory planning, Non-destructive testing, Inverse kinematics, Automotive inspection, Digital twin simulation, Engineering, Mathematics and computing, Physics

## Abstract

Computed tomography is a widely used imaging method for non-destructive testing. However, standard CT systems face fundamental limitations when scanning large objects such as car bodies, which must fit between the X-ray source and detector, both of which need freedom of movement around the specimen. Twin-robotic CT systems with high degrees of freedom address these limitations by enabling free positioning of the X-ray source and detector in space, making non-destructive CT testing of large objects feasible. However, achieving collision-free positioning of the robots is a challenging problem that is often neglected in theoretical representations of twin-robot CT configurations. This paper presents a systematic methodology for performing region-of-interest scans on large objects. The approach exploits the test object’s geometry to determine robot reachability, which serves as the foundation for trajectory planning by incorporating accessible regions. By leveraging both rotational and translational degrees of freedom, including variable source-detector distances and detector rotations, the methodology expands the range of collision-free poses, thereby increasing reachability and enabling more flexible trajectory design. The methodology is modular and adapts to arbitrary system configurations and test samples via computer-aided design (CAD)-based geometry definition, where the test object determines the collision-free workspace. It is demonstrated on a BMW 4-series body-in-white through comprehensive batch simulation across 273 Region of Interest (ROI) positions, evaluating reachability improvements achieved through the introduced degrees of freedom using a data completeness trajectory optimization criterion.

## Introduction

Computed Tomography (CT) is a widely used imaging method for non-destructive testing. It enables visualization of internal structures without damaging the sample. However, conventional laboratory CT systems have limited space to accommodate large objects such as car bodies, which must fit between the X-ray source and detector, both of which need freedom of movement around the specimen^1–3^. To scan large objects, either larger systems are built, requiring higher X-ray power and often compromising resolution^4^, or the specimen is segmented, which contradicts the principle of non-destructive testing.

To address these limitations, flexible systems such as robotic C-arm systems have been developed. These systems dynamically adjust the detector’s orientation and position, thereby increasing the effective Field of View (FoV). Rotating the detector to align its diagonal with the scan direction overcomes fixed-width limitations and enlarges the lateral FoV ^5^, enabling scanning of larger areas. Variable field-of-view techniques ensure proper sinogram coverage for non-circular areas^6^, while dynamic detector offsets reduce the required scan range for elliptical FoV, decreasing acquisition time^7^. However, C-arm systems remain restricted by their mechanical design to approximately circular trajectories, limiting their flexibility for object-specific scanning strategies.

To overcome trajectory limitations, single-robot CT systems have gained attention recently, where the specimen is manipulated to enable object-specific trajectories^8–11^. However, these systems cannot accommodate large components such as car bodies. Additionally, a single robot limits the effective number of degrees of freedom available for truly free trajectories^2,12–15^. Consequently, twin-robotic CT systems offer the most promising approach for scanning large objects with flexible trajectories.

**Fig. 1:**
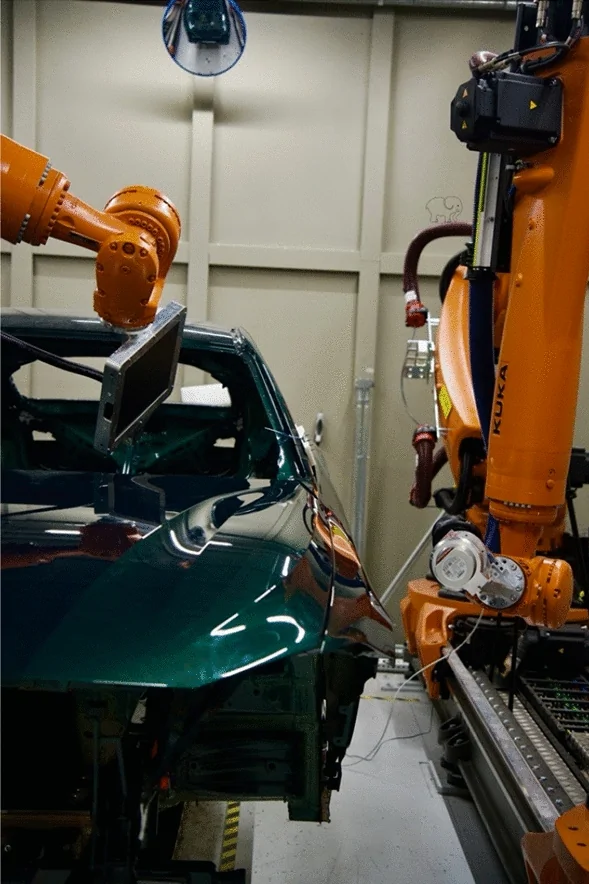
Twin-robotic CT system at the BMW Group Research and Innovation Center FIZ in Munich. The full system comprises four identical industrial KUKA robots. However, only the two robots shown in this figure are considered in this work, one carrying the X-ray source on the right robot arm and the other carrying the X-ray detector on the left robot arm. This configuration enables free CT trajectories around a given ROI of the car body (in the example the A-pillar).

Twin-robotic CT systems, in which both X-ray source and detector can be positioned freely around the specimen, address the limitations of alternative setups. In such systems, two industrial robots typically carry the X-ray source and detector respectively, enabling inspection of Regions of Interest (ROIs) on large components (see Fig. 1)^16^. The high number of degrees of freedom enables trajectory optimization tailored to specific inspection tasks, including region-of-interest scanning, data completeness maximization, and artifact reduction strategies that are infeasible with conventional circular trajectories^1,2,12,16–21^.

The high mobility of twin-robotic CT systems introduces substantial challenges, particularly for trajectory planning, collision avoidance, and handling robot-related inaccuracies. The literature contains numerous trajectory optimization approaches targeting various objectives such as minimizing X-ray path length to avoid beam-hardening and photon-starvation artifacts^12,22^, capturing edges as particularly informative projection directions^14^, maximizing quantitative measures of data completeness based on the Tuy-Smith condition^1,16,23^ or detectability indices^13,19,24^, or ensuring voxel-wise data completeness with detector offsets to guarantee accurate reconstruction^25^. These methods demonstrate how flexible detector trajectories can extend scan coverage by adapting to object geometry and system constraints. However, these methods generally assume idealized geometries and largely ignore actually reachable, collision-free robot poses. When systems are extended by additional degrees of freedom such as linear axes or lifting tables, these issues become even more pronounced. Consequently, a large fraction of prior work remains mostly theoretical. Critical aspects such as real reachability limits, calibration^26–29^, and collision checking^30,31^ are often treated only in simplified form or not at all. This is presumably one reason why free, object-specific trajectories for large components have not yet been demonstrated on a real twin-robotic system.

This work addresses part of this gap by incorporating collision checking directly into the trajectory design process, thereby narrowing the gap between theoretical trajectory optimization and practical feasibility. The proposed methodology explicitly accounts for collision constraints and robot kinematics based on CAD models, providing a practically grounded estimate of reachable viewing configurations. Specifically, we address three fundamental research questions:

1. How can collision-free reachability be determined and systematically increased by exploiting the kinematic degrees of freedom of twin robots together with the rotational and translational degrees of freedom of X-ray imaging geometry?
2. How can the determined reachable workspace be used as a foundation for trajectory optimization?
3. Which regions of a scan object can be scanned with optimized trajectories, and where are the system limitations?

This work presents a systematic methodology for determining reachable workspace based on CAD data. The core contribution addresses question (1) by maximizing collision-free reachability through exploitation of both the kinematic degrees of freedom of twin robots and the imaging degrees of freedom (rotation and translation) of X-ray geometry, which establishes the foundation for trajectory optimization. Implemented fully in simulation with CAD-based collision detection, the methodology adapts readily to new components and inspection tasks.

Questions (2) and (3) are addressed through comprehensive evaluation on a BMW 4-series body-in-white. The simulation environment replicates the twin-robotic CT system at BMW^3^. To demonstrate trajectory optimization within the determined reachable workspace, we employ data completeness based on the Tuy-Smith condition^23^ as a proof-of-concept criterion. This condition requires viewing each ROI point from at least 180$^\circ$ plus the source opening angle, ensuring data-complete reconstruction. While data completeness serves as the baseline optimization criterion in this work, the modular framework accommodates alternative criteria such as edge detection^14^, X-ray path length minimization^12,22^, or detectability indices^13,19^, and generalizes to arbitrary objects and system configurations through CAD-based geometry definition.

To answer these research questions, we conduct a comprehensive batch simulation across 273 ROI positions uniformly distributed over the car body’s right side. Four system configurations (coupled/uncoupled rotation *×* coupled/uncoupled orbital radii) are systematically tested at each position. This design enables us to (1) quantify how each degree of freedom affects workspace coverage, (2) identify regions accessible for data-complete scans, and (3) provide practical guidance on where data complete ROI scans are feasible.

This paper is structured as follows. The Methods section presents the reachability-maximization methodology and explains how rotational and translational degrees of freedom are exploited to systematically expand the collision-free workspace. The baseline data completeness criterion used for proof-of-concept trajectory selection is also introduced. The Experiments section describes the simulation setup, the BMW 4-series body-in-white test case, and the comprehensive batch evaluation methodology across 273 ROI positions. The Results section presents quantitative reachability improvements for different system configurations and demonstrates the identified optimal trajectories. The Discussion summarizes the findings, discusses limitations, and provides an outlook for future work. Finally, the Conclusion summarizes key findings and contributions.

## Methods

This paper presents a simulation-based methodology to determine the collision-free workspace for a twin-robotic CT system and to increase reachability by exploiting the rotational and translational degrees of freedom of X-ray imaging. Based on the reachable workspace, the methodology identifies optimal circular trajectories that maximize the data completeness required for CT scans. The methodology is implemented using ROS^32^ together with the MoveIt framework for inverse kinematics solving and collision checking. It uses CAD data of the scan object (a BMW 4-series car body) for collision monitoring. RViz is used for visualization of the simulation environment. The simulated system consists of two industrial robots, each with six degrees of freedom, one carrying the X-ray source and the other the detector.

**Fig. 2:**
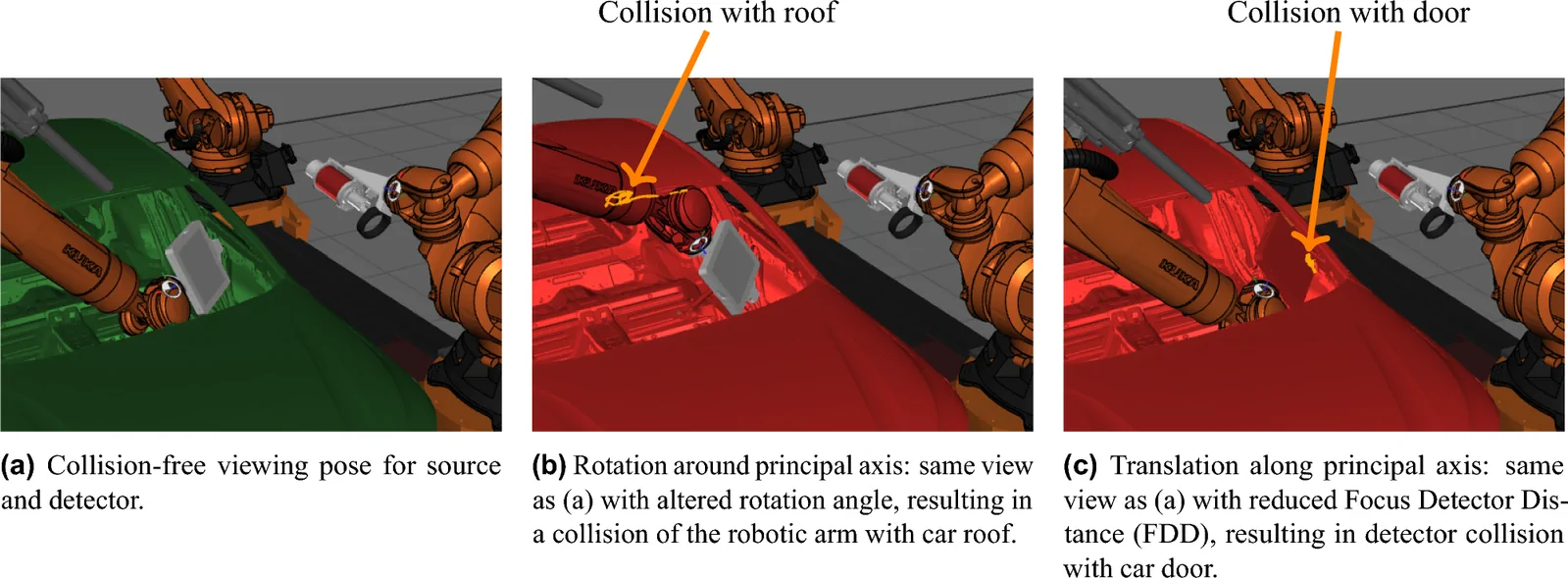
Illustration of how rotational and translational degrees of freedom affect collision-free reachability for a twin-robotic CT system. **(a)** A collision-free viewing pose $K_{inv}(\tilde{{\textbf {G}}_i})$ where both source and detector successfully reach their target positions. **(b)** An unreachable configuration obtained by rotating the detector around its principal axis $\vec {n}_z^{D}$ from pose (a), resulting in a collision with the car roof (orange lines show colliding CAD mesh, arrow indicates collision point). **(c)** An unreachable configuration obtained by translating the detector along its principal axis toward the scan object from pose (a), shifting to a smaller orbital radius and resulting in a collision with the car door (arrow indicates collision point). These examples demonstrate that exploiting rotational and translational flexibility is essential for maximizing workspace coverage while avoiding collisions.

The methodology is structured into several steps, where it is executed individually for each ROI. These steps are:

- **Basic Set of Potential Views.**
  A discretized set of potential views is distributed around the scan center point, where each view defines the position and orientation of the source and detector through transformation matrices. The distribution pattern can be adapted to the specific requirements of the inspection task.
- **Reachability Analysis.**
  For each potential view, it is verified whether a collision-free IK solution exists. Reachability is systematically increased by exploiting the rotational and translational degrees of freedom of the imaging geometry (see Fig. 2). Additionally, system-specific degrees of freedom can be integrated and evaluated within this framework.
- **Data Completeness Optimization.**
  Trajectory optimization is then performed on the set of reachable poses. In this work, we identify circular trajectories that maximize angular coverage to achieve data completeness, while minimizing scan time through selection of low orbital radii and coupled manipulator rotations.

The steps defined above are discussed in detail in the following.

### Basic set of potential views

The reachability analysis is performed within a complete digital twin of the system, incorporating kinematic models of both robots, CAD-based geometry of the scan object, and the robotic cell. Collision detection operates directly on these geometric models.

For physical implementation on a real twin-robotic CT system, reliable collision avoidance requires high fidelity between the digital twin and the physical setup, commonly referred to as twin closeness^33^. This is typically achieved by referencing the physical object’s position using measurement tools such as measurement rods to align the CAD model in the digital twin with millimeter-level accuracy^34,35^. Additionally, safety margins between robot and scan object can be defined in the digital twin to provide an additional layer of collision prevention.

To identify the optimal scan trajectory, a basic set of potential views is defined. This concept has been introduced in prior works^14,30,36^, and this study adopts a similar approach. For the definition of the basic set of potential views, we align with the formulation in^16^, where a potential view $\tilde{{\textbf {G}}}$ is defined as a pair of transformation matrices:

1 $$\begin{aligned} \tilde{{\textbf {G}}} := ({\textbf {T}}^S_W, {\textbf {T}}^D_W). \end{aligned}$$

Here, ${\textbf {T}}_W^{S}$ and ${\textbf {T}}_W^{D}$ denote the transformation matrices for the X-ray source ${\textbf {S}}$ and detector ${\textbf {D}}$, respectively, while ${\textbf {W}}$ represents the world coordinate system. Each transformation matrix ${\textbf {T}}_W^{S, D}$ is a *4 × 4* homogeneous matrix defined :

2 $$\begin{aligned} {\textbf {T}} = \begin{bmatrix} {\textbf {R}} & \vec {t} \\ \vec {0}^T & 1 \end{bmatrix}. \end{aligned}$$

**Fig. 3:**
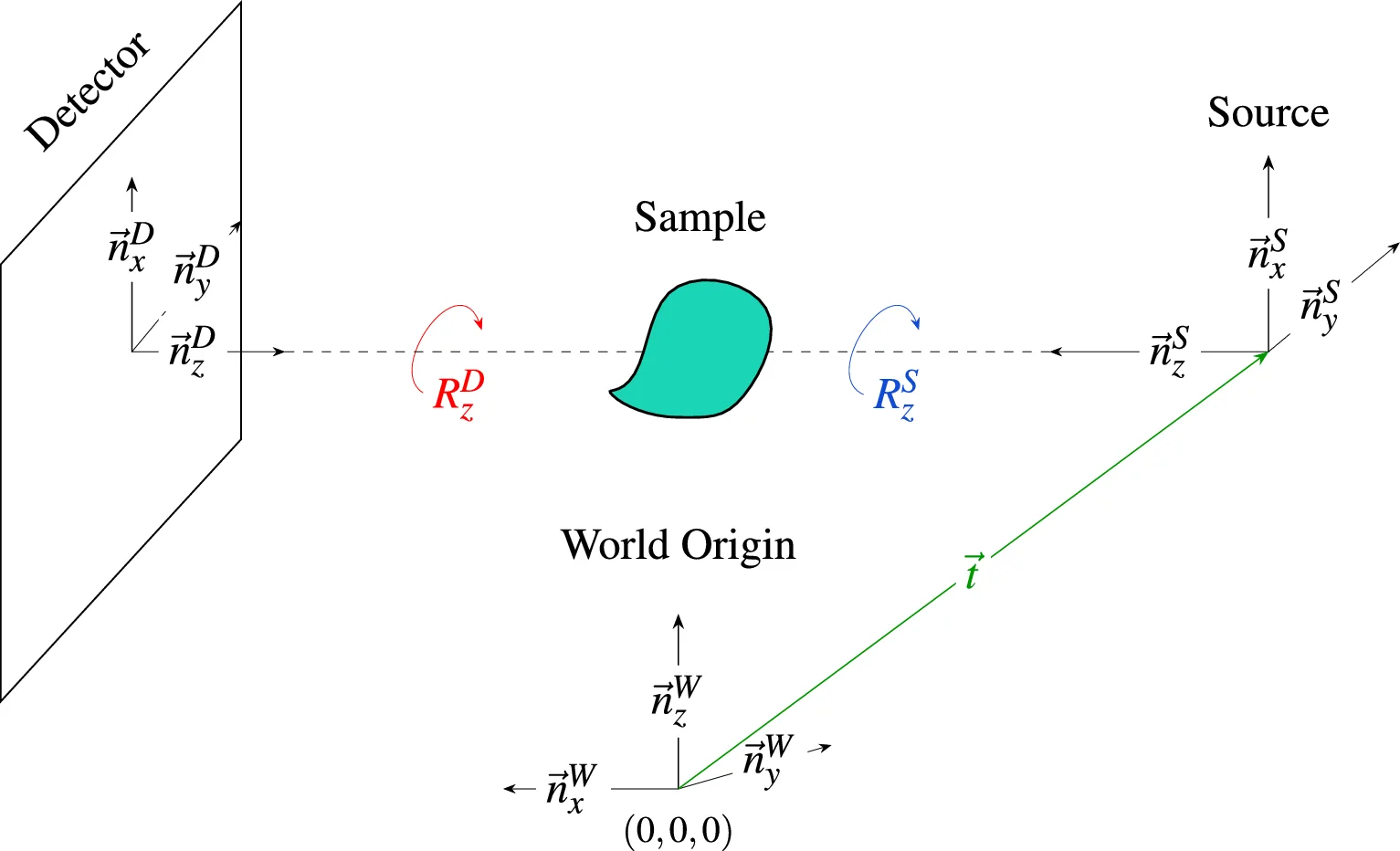
Illustration of the coordinate transformations from the world coordinate system to the manipulator coordinate systems, denoted as ${\textbf {T}}_W^{S}$ and ${\textbf {T}}_W^{D}$ for the source (S) and detector (D) in the world (W) coordinate system, respectively. The translational vector is represented by $\vec {t}$, while the normal vectors $\vec {n}^{S,D,W}_x$, $\vec {n}^{S,D,W}_y$, and $\vec {n}^{S,D,W}_z$ define the rotation matrices for the source (S), detector (D), and world (W) coordinate systems. The dashed line indicates the principal axis connecting the source and detector. A rotation $R_z^{S,D}$ around the principal axis $\vec {n}_z^{S,D}$ for both source and detector is shown, which increases the number of reachable robot poses without altering the imaging geometry.

In this equation, $\vec {t}$ is the translation vector specifying the position in world coordinates, and $\textbf{R}$ is the *3 × 3* rotation matrix, with $\textbf{R} = [\vec {n}_x, \vec {n}_y, \vec {n}_z]$, where $\vec {n}_x$, $\vec {n}_y$, and $\vec {n}_z$ are the orthonormal basis vectors (normal vectors) defining the orientation, as illustrated in Fig. 3. This formulation fully describes a potential view, comprising both the position and orientation of the X-ray source or detector in the world coordinate system.

A collection of such potential views defines the basic set of potential views. The geometric distribution of these views can be chosen in a task-specific manner depending on the scan object and inspection requirements. In this work, possible views are distributed using uniform spherical distributions around each ROI of the car body, similar to the approach in^30^. However, other geometric distributions such as cylindrical, planar, or custom patterns can be employed to enable different scanning strategies tailored to specific inspection tasks.

### Reachability analysis

For each potential view, the robot joint configuration is determined via IK solving. A pose $\tilde{{\textbf {G}}}$ is IK-reachable if at least one collision-free solution exists for both source and detector. Formally:

3 $$\begin{aligned} K_{inv}(\tilde{{\textbf {G}}_i}) = \{\vec {q}_{i,1}, \vec {q}_{i,2}, \dots , \vec {q}_{i,n_i}\} \ne \emptyset , \end{aligned}$$

where $K_{inv}$ maps Cartesian poses to feasible joint configurations $\vec {q}_{i,j}$, with *i* indexing the *s* reachable poses and *j* indexing the $n_i$ possible solutions for pose *i*.

Due to high robot degrees of freedom, multiple solutions may exist^16^. In this work, multiple IK solutions may be computed for each pose. A pose is classified as IK-reachable if at least one of these solutions is collision-free. Future work could exploit multiple solutions to optimize trajectory planning through selection of joint configurations that minimize motion, avoid joint limits, or improve smoothness. To verify reachability in practice, we use the MoveIt framework within ROS to compute IK joint configurations $\vec {q}_i$ and perform collision checking based on its planning scene representation (see Fig. 2a). Only if the robot is found to be collision-free in this pose is the pose classified as IK-reachable.

A trajectory $\tau _{Scan}$ describes the sequence of joint configurations the robot executes when moving between multiple viewing poses, and is defined in^16^ as:

4 $$\begin{aligned} \tau _{Scan} = \{\vec {q}_1, \vec {q}_2, \dots , \vec {q}_m\}, \end{aligned}$$

where *m* represents the number of poses in the scan trajectory. A trajectory is trajectory-reachable if collision-free motion plans exist connecting all consecutive poses. While IK-reachability verification scales linearly with the number of poses ($\mathcal {O}(n)$ complexity for *n* poses), trajectory-reachability requires motion planning between all pose pairs, resulting in $\mathcal {O}(n^2)$ complexity^37,38^. This makes exhaustive verification computationally prohibitive.

Since the aim of this work is to determine the optimal scan trajectory based on the reachable workspace, this methodology focuses on maximizing the density and spatial connectivity of IK-reachable poses around the scan object. A high density of IK-reachable poses increases the probability that collision-free paths may exist between neighboring poses^39–41^. Once the IK-reachability map is established, optimal trajectories can than be selected, and trajectory-reachability is subsequently verified only for these selected candidates. This approach ensures that computational resources are minimized while simultaneously guaranteeing high reachability. Since continuous collision-free motion between poses is not explicitly verified within this work, all trajectory results derived from the IK-reachability map should be interpreted as an upper bound on trajectory-reachability.

#### Methods to increase IK-reachability

IK-reachability is a crucial factor in ensuring collision-free and feasible robot poses in twin-robotic CT systems. Figure 3, in which the coordinate transformations are illustrated, provides the basis for the following discussion. To systematically increase IK-reachability, we employ two main strategies, which will be discussed in detail below. Figure 2 demonstrates how translation and rotation of the detector can result in collisions with the scan object. For each manipulator, both a translation vector $\vec {t}$ and a rotation matrix $\textbf{R} = [\vec {n}_x, \vec {n}_y, \vec {n}_z]$ are defined. Generally, the rotation is defined so that $\vec {n}_z^{S} = -\vec {n}_z^{D}$ holds, ensuring that the source and detector are always facing each other.

**Rotational degree of freedom:**

By allowing a rotation of the source and detector around their respective principal axis $R_z^{S,D}$ (see Figure 3), the range of reachable robot joint configurations can be expanded without changing the view information content relevant for reconstruction.

We distinguish two rotation modes. *Coupled rotation* synchronizes source and detector angular displacement around their principal axes, preserving relative imaging geometry. *Uncoupled rotation* allows independent rotation, expanding accessible workspace but requiring configuration-specific offset recalibration^16,31,42^. Independent rotations increase feasible positions and eliminate dependence on initial view configuration.

Rotations around additional axes ($R_x$ or $R_y$) could further enhance reachability and, when properly handled during reconstruction, improve spatial resolution through tilted detector geometries^43^. These are not considered in this work.

**Translational Degree of Freedom:**

By shifting the source or detector along their respective principal axes $\vec {n}_z^{S}$ or $\vec {n}_z^{D}$, the geometric configuration of the imaging system is altered. Specifically, this varies the Focus Detector Distance (FDD), Focus Object Distance (FOD), and Detector Object Distance (DOD), as illustrated in Fig. 3. These geometric parameters determine the magnification *M* according to:

5 $$\begin{aligned} M = \frac{FDD}{FOD} = \frac{FOD + DOD}{FOD} = 1 + \frac{DOD}{FOD}. \end{aligned}$$

Variable source-detector distances thus affect both the effective resolution of the scan and the reachable workspace. By adjusting these distances, additional viewing directions become accessible that would be unreachable at fixed distances^31^, thereby expanding the collision-free workspace (see Fig. 2c).

We distinguish two operational modes based on magnification behavior. In *coupled orbit* mode, the ratio between FOD and DOD remains constant throughout the scan, preserving magnification^31^. In *uncoupled orbit* mode, the FDD varies freely, resulting in magnification changes across the trajectory.

To maintain constant projection intensity during changes in FDD, the exposure time *t* must be adjusted during a scan to ensure a constant detector intensity *I* according to the inverse square law^31^:

6 $$\begin{aligned} t =\lambda (I) \cdot FDD^2 \end{aligned}$$

The parameter *λ (I)* represents a constant scaling factor determined by the desired detector intensity *I*. It is assumed to remain constant during the scan. Thus, any change in Focus Detector Distance requires a corresponding adjustment of the exposure time *t* to maintain constant detector intensity.

For physical implementation, each change in rotational or translational configuration during a scan necessitates additional offset-gain calibration of the detector to maintain image quality consistency across different geometric configurations.

#### Methodology to increase IK-reachability

These methods are combined into a systematic methodology for maximizing IK-reachability through iterative exploitation of rotational and translational degrees of freedom, balancing computational feasibility with comprehensive workspace coverage.

The methodology proceeds as follows:

- **Generate Basic Set of Potential Views:** Create a discretized set of potential views distributed around the scan point.
- **Check Reachability:** Verify whether collision-free IK solutions exist for both source and detector^1,16^.
- **Rotate:** Incrementally rotate source and detector around their principal axes and re-check reachability until maximum rotation is reached.
- **Translate:** Incrementally increase FDD to shift the orbit^31^ and re-check reachability until maximum orbital radius is reached.
- **Additional DOFs:** Systematically adjust system-specific degrees of freedom (e.g., linear axis positioning, lifting table height, rotation stage angle) and return to step 2 to check reachability. Continue until all available degrees of freedom are exhausted.

The iterative process continues until all sampled configurations of the considered degrees of freedom have been evaluated, resulting in the complete IK-reachable space within the chosen discretization.

#### Workspace coverage

For the evaluation, we define workspace coverage as the percentage of distinct viewing directions from the basic set of potential views that can be realized for a given ROI. Workspace coverage describes what proportion of unique viewing angles on spheres with variable radii around the ROI can be reached in a collision-free manner. Importantly, we count only poses that provide new viewing directions. If a pose at a larger orbital radius offers the same viewing direction as a pose at a smaller radius, it is not counted as an additional viewing angle. This quantifies the angular diversity of accessible viewing directions rather than the total number of reachable poses. A workspace coverage of 100% represents the ideal case where all viewing directions from the basic set of potential views are reachable.

### Data completeness

Following IK-reachability determination, trajectories are optimized for data completeness. In this paper, we employ a baseline data completeness criterion defined as radial coverage of at least 180$^\circ$ plus the source opening angle^22,23,31^. This criterion enables accurate reconstruction when combined with Parker weights^44–46^. Trajectories with incomplete coverage can be addressed through compensation weights combined with bilateral filtering or total variation regularization^47^.

The data completeness criterion serves as a proof-of-concept for trajectory optimization within the reachable workspace. While more refined analyses could account for the detector’s active area and its variation with FDD, the simplified criterion effectively demonstrates the methodology’s capability to identify feasible scan trajectories. The reachability maximization framework provides a foundation applicable to any trajectory optimization criterion, including more sophisticated data completeness metrics.

An additional optimization objective is to minimize total scanning time. Within the considered setup, scanning time is predominantly determined by the exposure time required for each image acquisition, as robot motion between closely spaced poses on a circular trajectory is assumed negligible. According to Equation 6, total scanning time can be reduced by operating at the lowest feasible orbital radius. Hence, the optimization aims to identify the longest trajectory at the lowest orbital radius.

From the IK-reachable workspace, we identify circular trajectories composed of multiple joined arcs. Trajectory length is defined as the angular span of this circular path. Trajectories exceeding 220$^\circ$ are classified as data-complete (satisfying the baseline criterion), while 360$^\circ$ trajectories form closed circles. For each identified optimal trajectory, poses are resampled with finer spatial resolution and validated for collision-free reachability, accounting for rotational degrees of freedom of both source and detector systems.

## Experiments

The methods described above are applied to the simulation of a twin-robotic CT system for scanning a car body. While no physical scan is performed in this study, the simulated system closely replicates the actual setup of the twin-robotic CT system located at BMW^3^. The real system comprises four industrial robots of type KUKA KR90 R3100 extra HA^48^, including two robots equipped with X-ray sources, one robot carrying the detector, and one robot equipped with an optical tracking system. For the simulation, only a single robot pair for X-ray imaging is considered.

The simulated system comprises two KUKA KR90 R3100 extra HA robots^48^, each with six degrees of freedom, positioned symmetrically at ±2.0 m along the y-axis and 0.7 m height (z-axis). The linear axis provides translational positioning along the x-axis, evaluated at four discrete positions (2.0 m, 3.25 m, 4.5 m, and 5.75 m) as an additional system-specific degree of freedom. Here, 5.75 m corresponds to the physical travel limit of the linear axis, while 2.0 m represents a feasible configuration within the accessible workspace. The X-ray source is modeled as a Comet Vario Focus 225 kV^49^, and the detector as a Varex XRD-3025^50^. The test object is a BMW 4-series body-in-white positioned at a constant height of 0.6 m. This physical setup is replicated in simulation using ROS 2 (Humble) together with the MoveIt framework for inverse kinematics solving and collision checking, enabling the evaluation of IK-reachability.

The objectives of these experiments are:

- quantify how the coverage area for robot reachability can be systematically enhanced by exploiting both rotational and translational degrees of freedom.
- identify regions suitable for region-of-interest (ROI) scans through trajectory optimization based on data completeness criteria.

Therefore, we evaluate different poses across the car body. The distribution and definition of these evaluation poses are described in the following subsection.

### Batch simulation

**Fig. 4:**
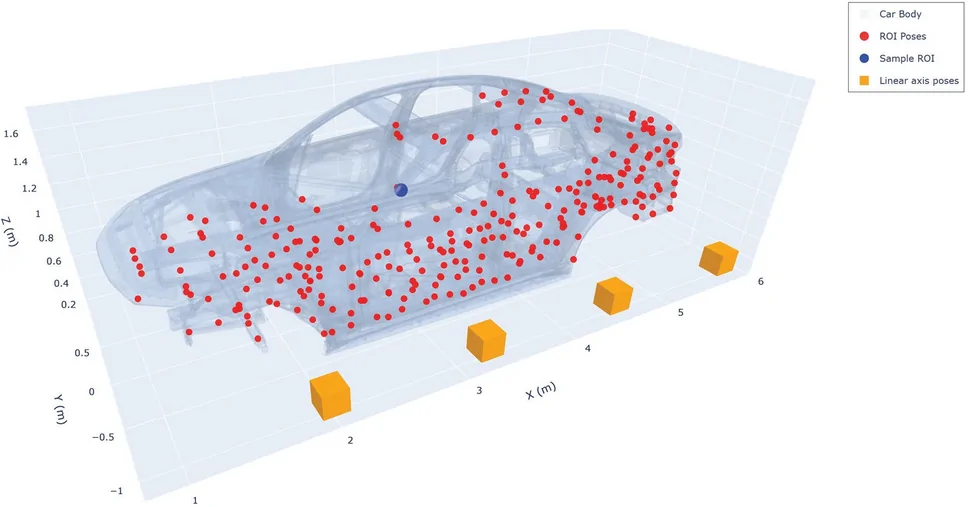
Distribution of the scan center poses over the left side of the BMW 4-series car body. Due to symmetry, the evaluation is performed only on the left side of the car body with dimensions of 4794 mm (length) *×* 1887 mm (width) *×* 1442 mm (height). Each of the 273 poses (red points) serves as the center for a spherical basic set of potential views used in the IK reachability analysis and data completeness framework. The blue point at the A-pillar highlights the sample point at which the analysis methodology is subsequently explained and demonstrated. The orange squares indicate the four different linear axis poses of the robots.

Due to vehicle symmetry, the ROI scan center poses are uniformly distributed over the right side of the BMW 4-series car body with 20 cm spacing between consecutive poses to ensure complete coverage (see Fig. 4). The IK-reachability results can be directly transferred to the left side by mirroring the source-detector configuration. This corresponds to a physically realizable arrangement in the twin-robotic CT system, where symmetric configurations exist on opposing sides of the setup.

Given the field of view of the CT system, this spacing ensures that each part of the vehicle can be evaluated for CT scanning capability. This distribution yields 273 ROI poses that are analyzed in the batch simulation. Each ROI pose is evaluated using the methodology described in Section Reachability Maximization Methodology. For this simulation, the car body is positioned at a fixed height of 0.6 m, and the lift table remains stationary.

For each batch, a reachability analysis is performed around each scan center pose using a basic set of potential views. For this purpose, a spherical distribution of possible views is generated with 2500 uniformly distributed poses on each sphere.

Source and detector orientations are constrained to face each other during spherical pose generation, ensuring $\vec {n}_z^{S} = -\vec {n}_z^{D}$. Orbital radii vary from 100 mm to 900 mm in 100 mm increments. The 900 mm upper limit corresponds to a 1800 mm maximum transmission path length, representing the assumed practical penetration limit for the 225 kV, 16 kW X-ray source through steel-aluminum composite structures. Additional constraints accounting for detector geometry and field of view^7^ could be incorporated (Section Data Completeness) but lie beyond this work’s scope. Rotational sampling uses 12 steps at 30$^\circ$ increments (0$^\circ$–330$^\circ$). For each ROI position, 2,500 base view poses are combined with 12 rotational configurations and 9 orbital radii, resulting in 270,000 candidate views tested for IK-reachability per ROI pose.

To analyze the influence of linear axis positioning, each of these 273 ROI poses was additionally evaluated at four discrete positions along the x-axis (2.0 m, 3.25 m, 4.5 m, and 5.75 m, as indicated in Fig. 4). The maximum position of 5.75 m corresponds to the physical travel limit of the linear axis in the BMW twin-robotic CT system, while the lower bound of 2.0 m was selected as a representative, feasible configuration within the accessible workspace.

### Reachability analysis

In the following, the results are presented exemplarily for the ROI position at the A-pillar (blue point in Fig. 4) using the uncoupled rotation and uncoupled orbit configuration. The results for the remaining configurations were evaluated analogously using the methodology described here and are subsequently assessed globally across all ROI positions.

**Fig. 5:**
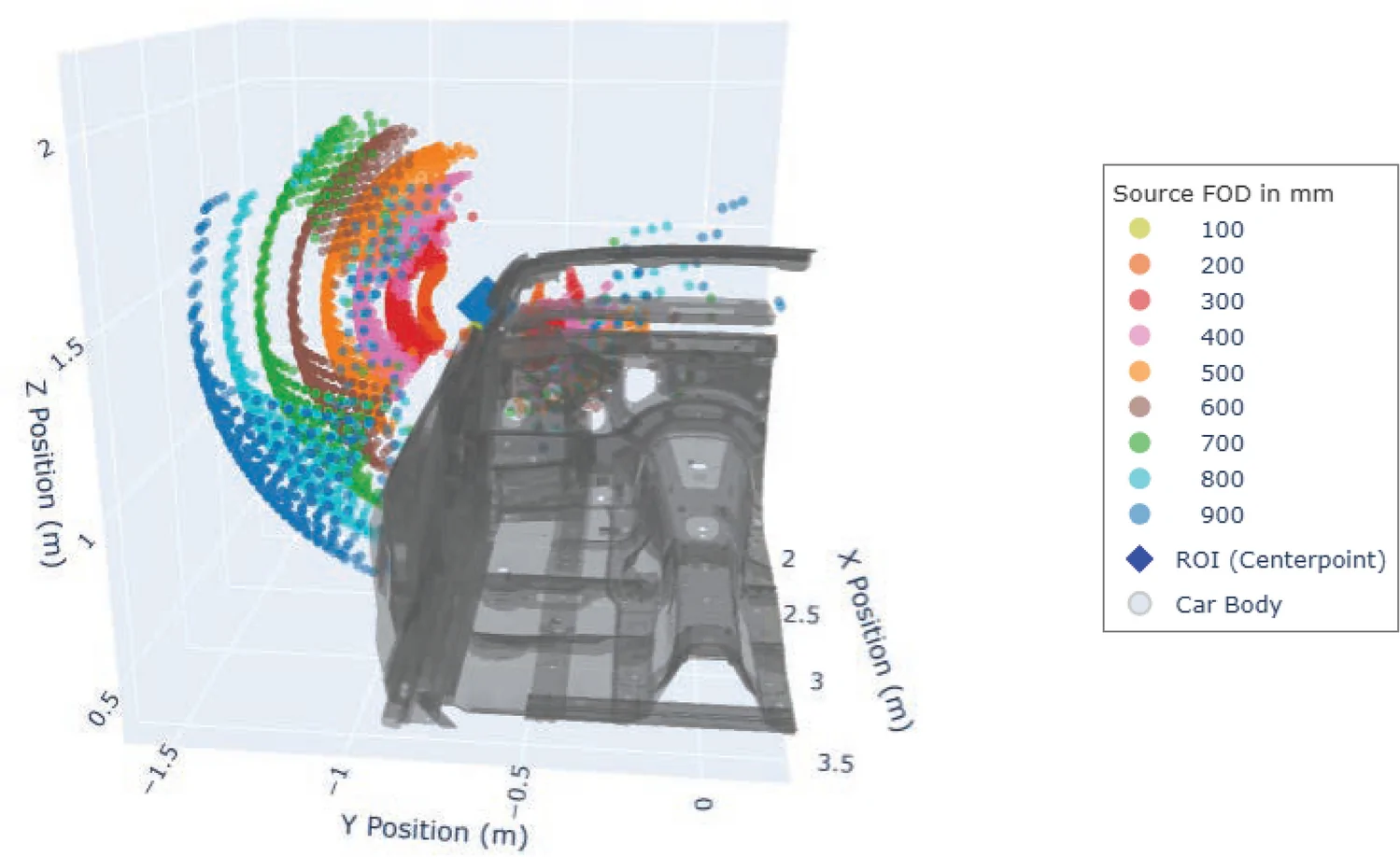
IK-reachable source poses at the A-pillar (marked in blue in Fig. 4) region for the uncoupled rotation and uncoupled orbit configuration. For visualization clarity, only source positions are shown while corresponding detector positions on the opposite side are not shown. The colors of each viewing indicate the corresponding orbital radius (100–900 mm).

The basic set of potential views undergoes IK reachability evaluation using MoveIt framework within ROS collision detection, as described in Section Reachability Maximization Methodology. Rotational and translational degrees of freedom are exploited to maximize the set of collision-free positions. Figure 5 visualizes the resulting workspace with orbital radii color-coded and the ROI center marked by a blue rectangle. These poses establish the foundation for trajectory optimization.

**Fig. 6:**
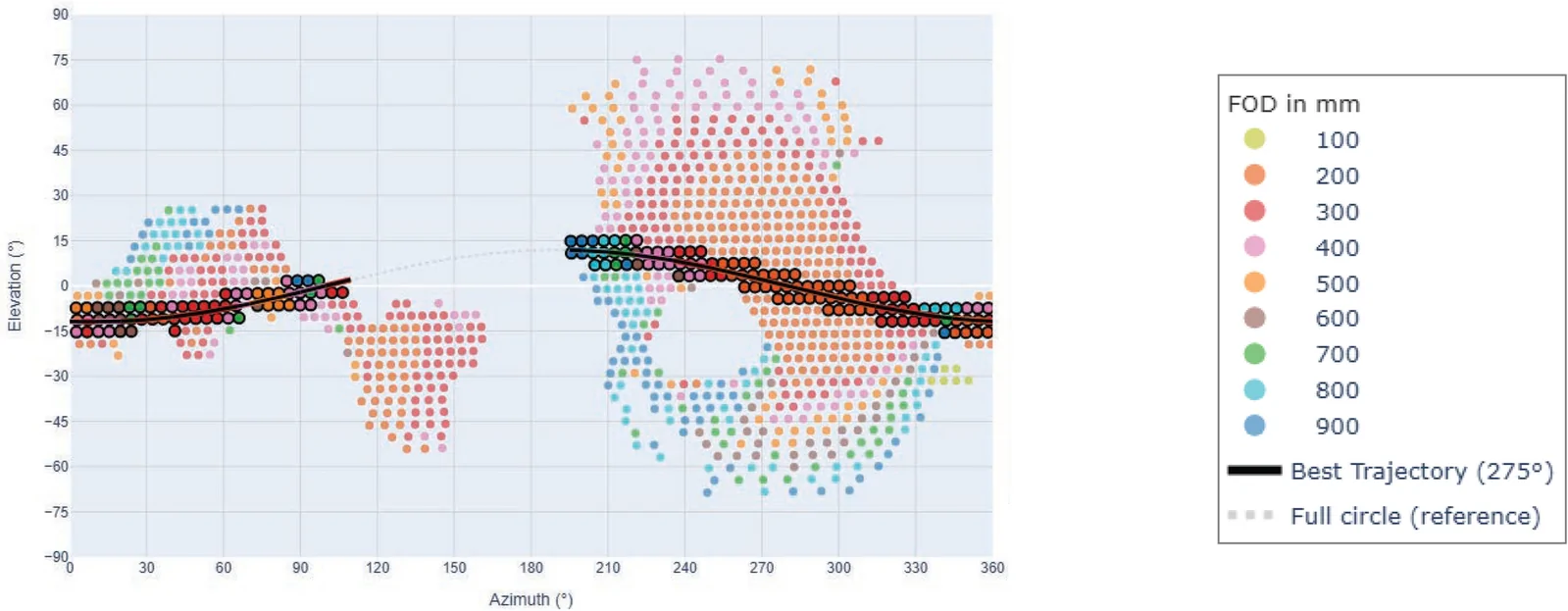
Azimuth-elevation plot of IK-reachable poses at the A-pillar (marked in blue in Fig. 4) region for the uncoupled rotation and uncoupled orbit configuration. The 3D poses from Fig. 5 are shown in this plot in their spherical coordinates. The radial components are color-coded (100–900 mm), with each viewing direction displayed at its minimum achievable orbital radius, ensuring that directions reachable at multiple radii appear only once. The plot also shows the optimized longest trajectory (black line), which achieves a data-complete trajectory of 275$^\circ$ for this scan. The corresponding spherical radius points for this trajectory are highlighted.

Figure 6 displays the reachable positions from Fig. 5 in spherical coordinates (azimuth and elevation angles). Starting from the smallest orbital radius, all positions with distinct viewing directions are shown, with colors indicating the minimum achievable orbital radius for each direction.

**Fig. 7:**
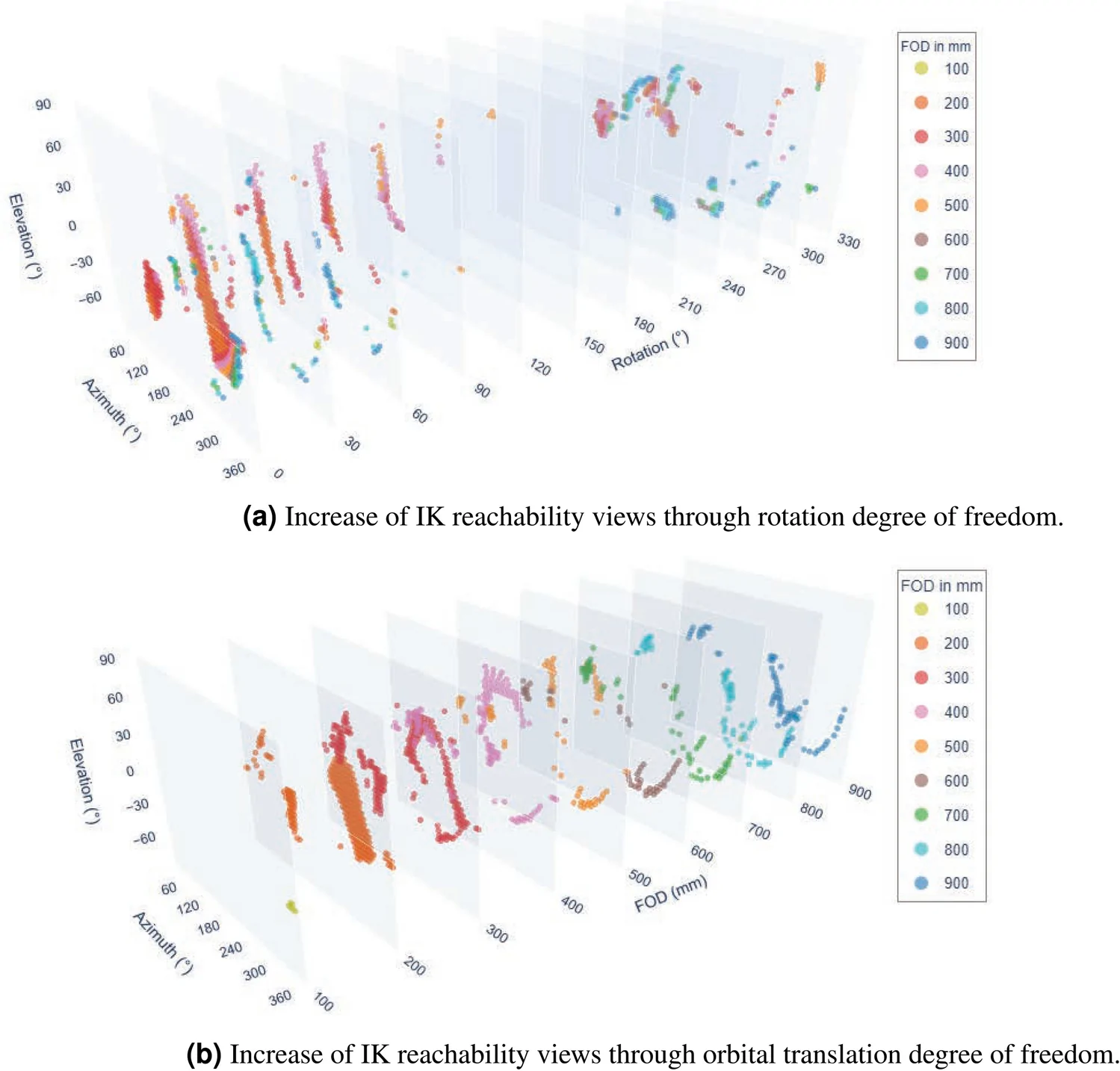
Reachability increase for the ROI at the A-pillar (marked in blue in Fig. 4). This figure shows IK-reachable poses in spherical coordinates for the uncoupled rotation and uncoupled translation configuration, evaluated at four linear axis positions. Each viewing direction is displayed at its minimum achievable orbital radius and color-coded accordingly. **(a)** Incremental reachability gain from rotational degrees of freedom. Each layer represents the azimuth-elevation distribution after adding an additional rotation angle. **(b)** Incremental reachability gain from translational degrees of freedom. The depth axis represents increasing orbital radii (100–900 mm), showing which new viewing directions become accessible at larger radii. By systematically exploiting both rotational and translational degrees of freedom, the complete reachable workspace is obtained, as shown in the azimuth-elevation plot in Fig. 6.

Figure 7 illustrates how different degrees of freedom contribute to workspace expansion. Subfigure 7a demonstrates the reachability increase achieved through rotational degrees of freedom. Additional rotation angles increase the sampling density of viewing directions, filling gaps within already-accessible regions. Subfigure 7b shows the reachability increase through translational degrees of freedom. Variable orbital radii primarily expand coverage at the boundaries of connected regions.

The populated azimuth-elevation plot with the maximized reachability is then used for trajectory optimization. We then identify the longest continuous arc segment that satisfies the data completeness criterion as a base trajectory optimization method. By design of the plot, the lowest possible orbital radius is selected for each view, which minimizes scanning time according to Equation 6.

**Fig. 8:**
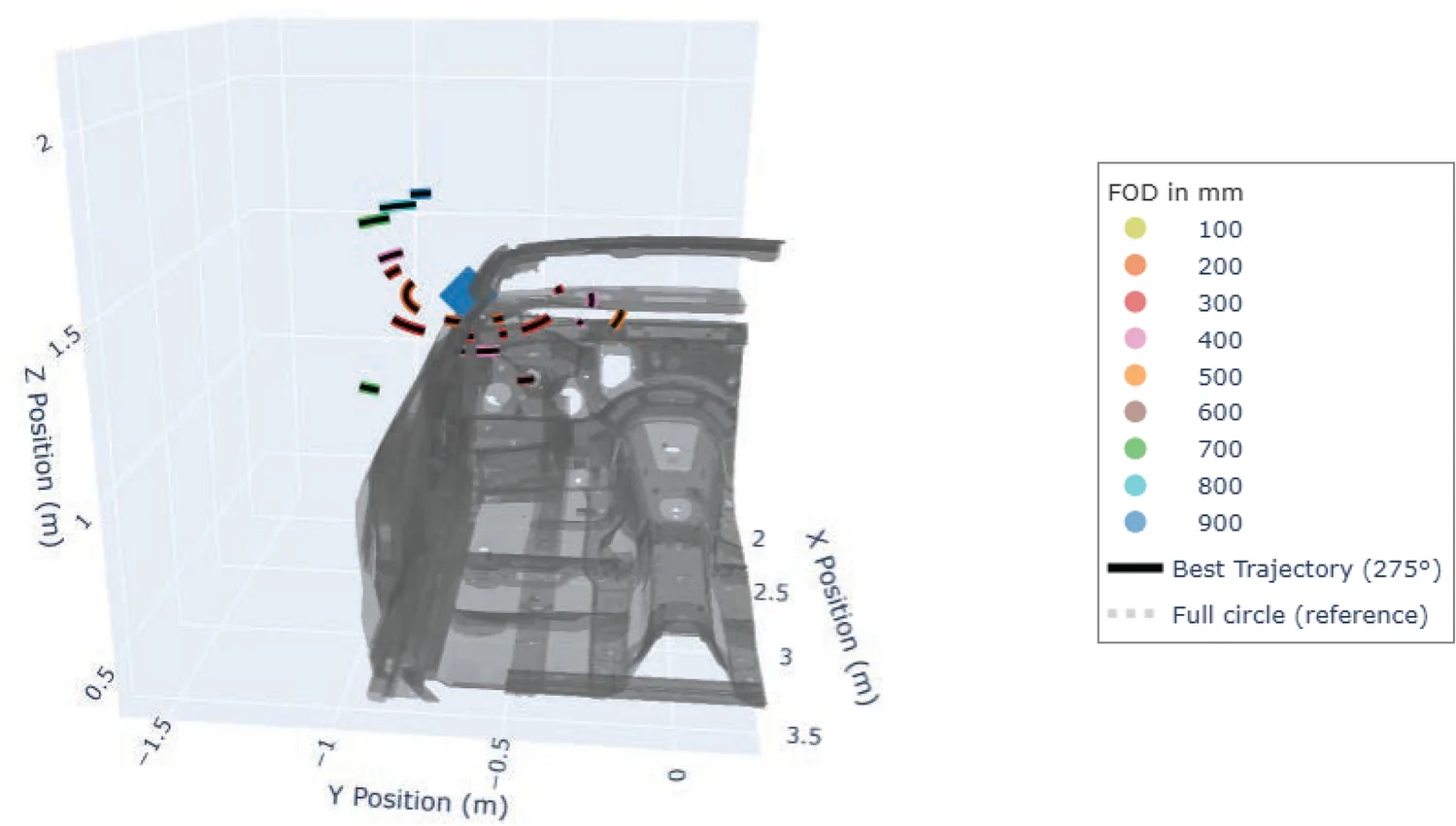
Optimized trajectory for the A-pillar ROI (same trajectory as shown in Fig. 6) achieving 275$^\circ$ arc length, which exceeds the 220$^\circ$ data completeness criterion. The trajectory is selected from the reachable workspace for the uncoupled rotation and uncoupled orbit configuration at 2.0 m linear axis position.

For the ROI at the A-pillar with uncoupled rotation and uncoupled orbital radius, we achieve a circular segment of 275$^\circ$, which satisfies the data completeness criterion of 220$^\circ$ (180 $^\circ$ plus 40 $^\circ$ opening angle of X-ray source). The resulting optimal trajectory is shown in Fig. 8.

This procedure is applied analogously to all 273 positions across the entire car body. In the following, we present the global validation results for the baseline configuration (coupled rotation and coupled orbit), which serves as the reference against which all other configurations are benchmarked.

**Fig. 9:**
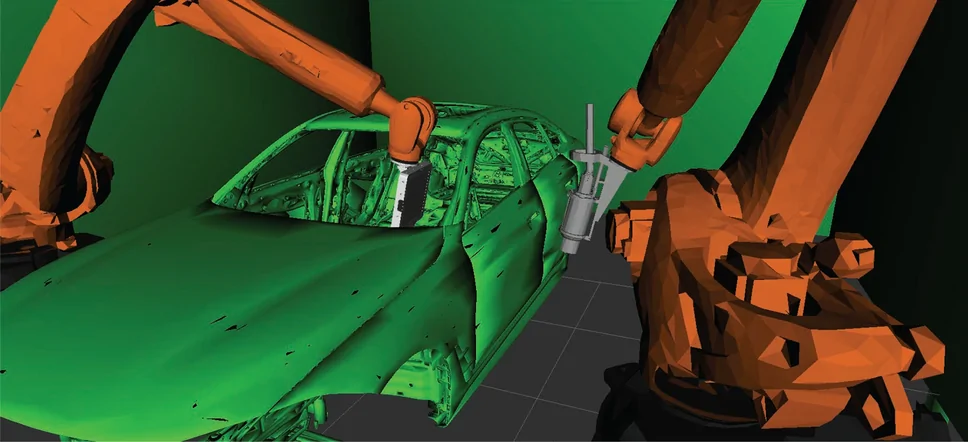
Simulation environment from RViz showing the standard starting orientation of the X-ray source and detector, with the ROI positioned at the hood of the BMW 4-series car body.

This configuration represents the most constrained scenario and demonstrates the foundation upon which the proposed methodology builds to increase reachability. Coupled rotation configurations are highly sensitive to the initial source-detector orientation. To ensure fair comparison across all experiments, we use the same standard starting configuration for all simulations (see Fig. 9). Manual optimization of the initial orientation for each ROI position would improve baseline performance but contradicts the methodology’s purpose: to systematically determine optimal configurations through automated exploitation of rotational and translational degrees of freedom. Furthermore, the coupled rotation configuration represents a subset of the orientations evaluated in the uncoupled case, where all rotation angles are systematically sampled. Consequently, the uncoupled configuration inherently includes the optimal starting orientation, effectively removing the dependency on the initial configuration by design.

In the following, we use the coupled rotation and coupled orbit configuration as a baseline to benchmark the reachability improvements achieved by the other configurations.

## Results

The methodology was designed to systematically evaluate how exploiting rotational and translational degrees of freedom enhances workspace coverage for robot reachability and identifies regions suitable for ROI scans with optimized trajectories. To assess this, we compared the performance of the proposed methodology against the baseline configuration (coupled rotation and coupled orbit) across 273 ROI positions distributed over the BMW 4-series car body.

The computations were performed on a CPU-based system (dual Intel®Xeon®, 64 cores) without specific optimization. On average, the evaluation of a single ROI with 270,000 views requires approximately 3 minutes per linear axis position, corresponding to approximately 14 hours for all ROI and approximately 50–60 hours in total across all four linear axis positions.

The analysis focused on two key aspects:

- the quantification of workspace coverage increase through rotational and translational degrees of freedom,
- the increase of areas where we can perform data-complete trajectories.

For the evaluation, we employ two metrics defined in Section Methods. Workspace coverage quantifies the percentage of distinct viewing directions from the basic set of potential views that are IK-reachable at each ROI position. Trajectory length measures the angular span of the longest circular trajectory composed of joined arcs, with trajectories exceeding 220$^\circ$ classified as data-complete. The derived trajectories are obtained from the map of IK-reachable poses and therefore do not guarantee trajectory-reachability. Consequently, the reported trajectory lengths should be interpreted as an upper bound on achievable trajectories. However, the methodology is designed to generate densely connected regions of IK-reachable poses, which increases the likelihood that collision-free motion between neighboring poses exists^39–41^. At the same time, this approach avoids the need for exhaustive pairwise motion planning, thereby significantly reducing computational complexity. As such, the identified trajectories provide a practically meaningful estimate of feasible scan trajectories while maintaining computational tractability.

**Fig. 10:**
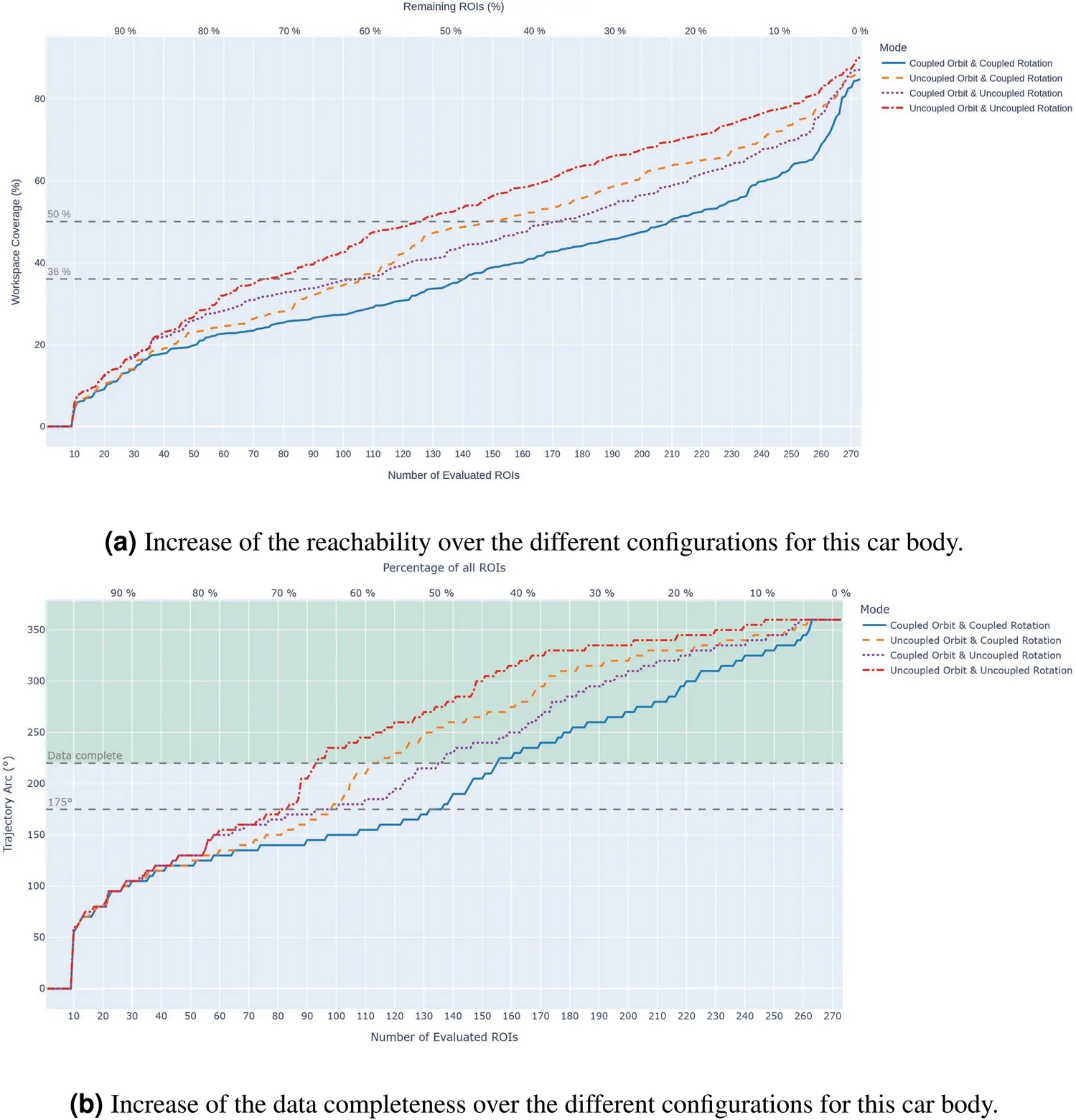
Quantitative comparison of reachability improvements across four configurations for the BMW 4-series car body, evaluated over 273 ROI positions and four linear axis positions. For both plots, the lower x-axis shows the ROI positions sorted in ascending order for each configuration independently. Each configuration is represented by a colored curve, which can be interpreted as a cumulative distribution. The upper x-axis shows the corresponding percentage of ROI positions in descending order. **Subfigure (a):** The y-axis shows the percentage of reachable viewing directions per ROI. For example, for the uncoupled orbit and uncoupled rotation configuration, approximately 55% of all ROI positions achieve at least 50% workspace coverage. **Subfigure (b):** The y-axis shows the maximum achievable circular trajectory length per ROI. The dashed line indicates the data completeness threshold of 220$^\circ$. For example, for the uncoupled orbit and uncoupled rotation configuration, approximately 66.3% of all ROI positions achieve data-complete trajectories.

In Fig. 10, we plot the cumulative coverage distribution for all evaluated poses across the different configurations. The analysis reveals substantial improvements in both workspace coverage and trajectory reachability across the evaluated configurations relative to the coupled rotation and coupled orbit baseline. The combination of uncoupled rotation and uncoupled orbit yields the highest workspace coverage.

The cumulative distribution curves in Subfigure 10a reveal distinct performance characteristics across configurations. At a workspace coverage threshold of 50%, approximately 55% of the evaluated ROI poses achieve this level with the uncoupled rotation and uncoupled orbit configuration (red dashed line), while the coupled rotation and coupled orbit baseline achieves this for only approximately 25% of evaluated ROI positions. The intermediate configurations show intermediate performance, where the uncoupled orbit with coupled rotation achieves 50% coverage at approximately 45% of poses, while coupled orbit with uncoupled rotation achieves this at approximately 35% of poses.

Across all coverage levels, the uncoupled orbit and uncoupled rotation configuration outperforms the other configurations, while the coupled rotation and coupled orbit baseline configuration (blue line) consistently shows the lowest performance. An interesting crossover behavior occurs between the uncoupled orbit with coupled rotation (yellow line) and the coupled orbit with uncoupled rotation (purple line). Up to approximately 36% workspace coverage, the uncoupled orbit with coupled rotation performs better. Beyond this threshold, the coupled orbit with uncoupled rotation outperforms it, maintaining this advantage up to nearly 80% coverage, where the relationship changes again.

Subfigure 10b presents the cumulative distribution of optimized trajectory arc lengths achieved across all ROI positions. Once again, the totally uncoupled configuration outperforms all other configurations across the entire range of arc lengths.

The uncoupled rotation and uncoupled orbit configuration (blue line) achieves the data completeness criterion of 220$^\circ$ arc length at 66.3% of all evaluated positions, while the coupled baseline (red dash-dot line) reaches this threshold at only 43.6% of positions. The coupled baseline configuration and the coupled orbit with uncoupled rotation (purple line) both exhibit an approximately linear increase in achievable arc lengths beyond 220$^\circ$. In contrast, the uncoupled rotation with uncoupled orbit and the uncoupled orbit with coupled rotation (yellow line) show a much steeper increase in this region, indicating that these configurations expand the set of positions capable of supporting longer trajectories more rapidly. For arc lengths below approximately 175$^\circ$, uncoupled rotation appears to be the dominant degree of freedom enabling longer trajectories. In this region, the coupled orbit with uncoupled rotation configuration (purple line) performs nearly identically to the total uncoupled configuration, suggesting that rotational flexibility alone is sufficient for achieving moderate arc lengths.

**Fig. 11:**
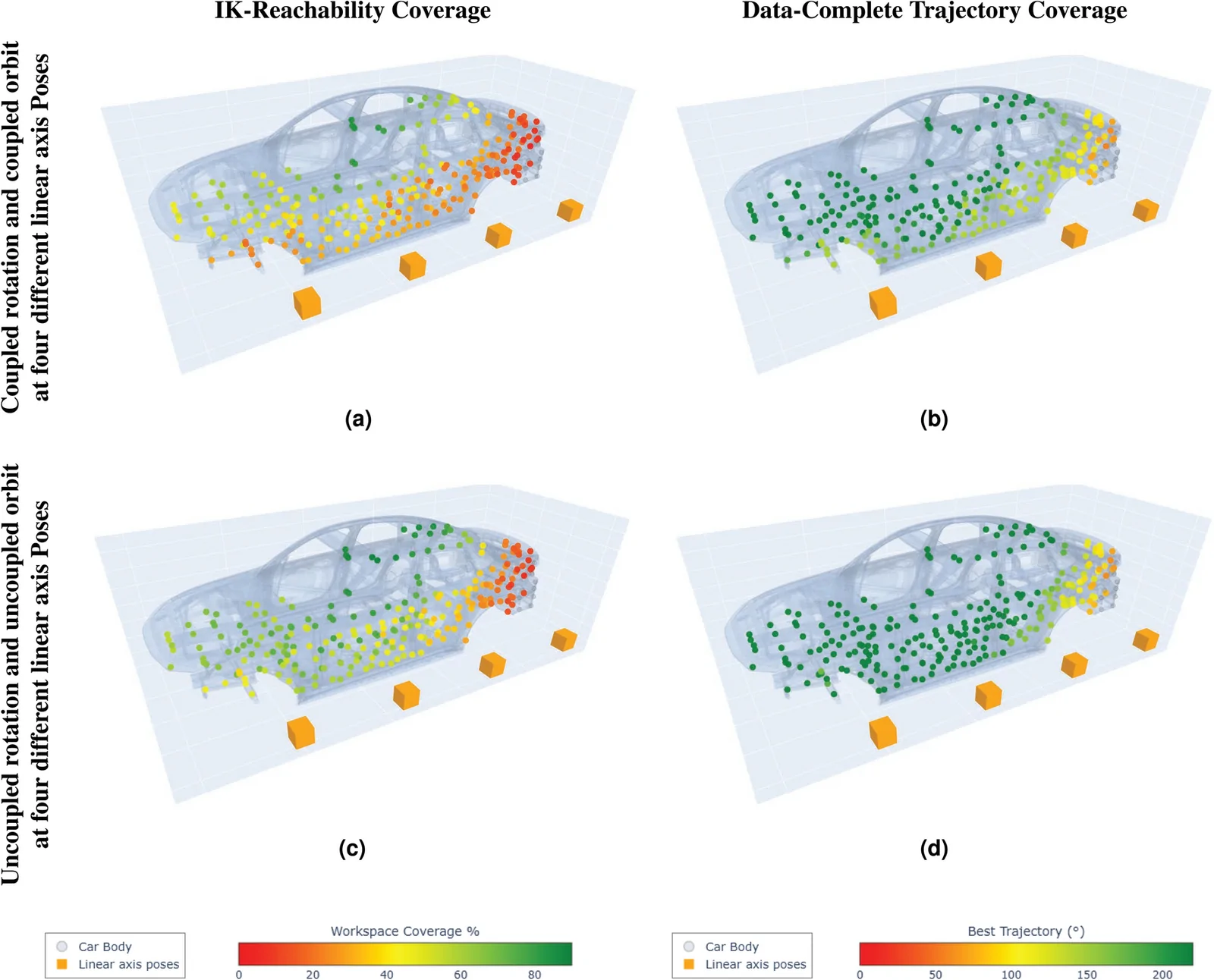
Comparison of baseline and optimized configurations across the BMW 4-series car body from Figure 4. **Left column:** IK-reachability workspace coverage showing the percentage of candidate poses achieving collision-free solutions at each ROI position (color-coded from red/low to green/high, gray indicates unreachable positions). **Right column:** ROI positions enabling data-complete trajectories with angular coverage exceeding 220$^\circ$ (green markers indicate scannable regions). **Top row (a–b):** Coupled rotation and coupled orbit baseline configuration. **Bottom row (c–d):** Uncoupled rotation and uncoupled orbit configuration demonstrating substantial improvements in both workspace coverage.

Figure 11 presents a direct spatial comparison between the baseline coupled rotation and coupled orbit configuration (Subfigures 11a and 11b) and the uncoupled rotation and uncoupled orbit configuration (Subfigures 11c and 11d). As discussed above, the uncoupled configuration achieves substantially higher coverage (51.8%) and data-complete trajectory reachability (66.3%) compared to the coupled configuration (37.8% and 43.6%). Three distinct patterns emerge from this spatial comparison:

- **Workspace Coverage distribution:** The coupled baseline (Subfigure 11a) shows high reachability (green regions) concentrated in the front rooftop region, while the uncoupled configuration (Subfigure 11c) achieves more uniform coverage extending into interior, floor, and front regions.
- **Data-complete trajectory distribution:** The coupled baseline achieves data-complete trajectories (green markers, Subfigure 11b) at 119 of 273 positions (43.6%), concentrated on the hood, roof, and front door exteriors. The uncoupled configuration extends this to 181 positions (66.3%), adding substantial coverage at the rear door transition zones and interior floor regions. This expansion demonstrates that difficult-to-reach poses become accessible through the systematic exploitation of rotational and translational degrees of freedom.
- **Reach limitations:** Both configurations show unreachable positions (gray markers) in the rear taillight region, where the detector robot would need to extend over the entire car body, exceeding its maximum reach. While car body reorientation could address this constraint, extending linear axis travel beyond the current 5.75 m limit is not physically possible within the evaluated BMW system’s workspace boundaries. This represents a fundamental system limitation rather than a methodology limitation.

**Fig. 12:**
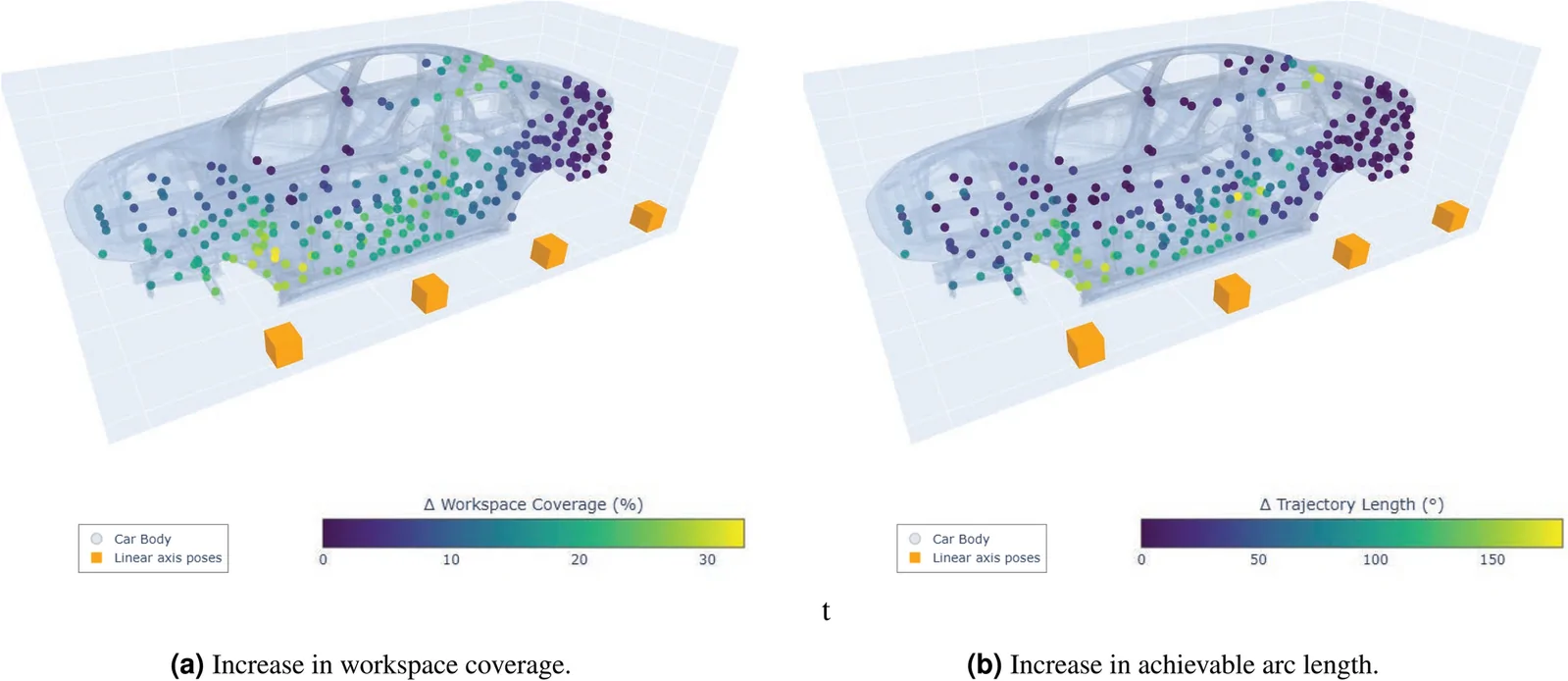
Spatial distribution of reachability improvements for uncoupled rotation and uncoupled orbit configuration compared to coupled baseline, for the car body from Fig. 4. **(a)** Percentage increase in IK-reachable workspace coverage across the car body (yellow: 33% improvement), showing substantial gains especially in floor regions. **(b)** Improvement in achievable longest circular arc length (yellow: 180$^\circ$ improvement). Both visualizations demonstrate that difficult-to-access regions benefit most substantially from the additional degrees of freedom.

Figure 12 presents the spatial distribution of reachability improvements for the uncoupled rotation and uncoupled orbit configuration compared to the coupled baseline configuration.

Subfigure 12a shows the increase in workspace coverage distribution by presenting the change in workspace coverage from the coupled baseline configuration compared to the complete uncoupled configuration. This visualization demonstrates substantial improvements in the front region. Additionally, improvements are observed in interior regions where the robots must move into the car body structure. This underscores that difficult-to-access regions particularly benefit from the increased reachability.

Subfigure 12b presents the change in trajectory length from the baseline to the complete uncoupled configuration. Here we observe an increase in achievable circular arc segments enabled by the expanded workspace discussed before. The results reveal regions that benefit substantially from the enlarged workspace, achieving improvements up to 180$^\circ$ at the transition region from the windshield to the steering wheel area. The trajectory arc length improvements reveal that 62 positions gain sufficient reachability in the uncoupled configuration compared to the baseline configuration, enabling transitions from non-data-complete ($<220^\circ$) to fully data-complete ($\ge 220^\circ$) trajectories. These positions are distributed mainly at floor-to-engine room, door-to-floor, and back door regions, where the expanded workspace now enables data-complete circular paths. Furthermore, only very few positions show no benefit from this expanded workspace, mostly at the rear part of the car body. The poses in the front and rooftop regions that exhibit minimal improvement from the enhanced reachability had already achieved near-maximal trajectory lengths (approximately 300$^\circ$) in the coupled baseline configuration (Subfigure 11b), indicating that these regions were operating close to their geometric upper bounds and thus offered limited potential for further optimization.

As a final result, Table 1 quantifies the reachability improvements across all four configurations as well as for the four cumulative linear axis positions. The uncoupled rotation and uncoupled orbit configuration consistently achieves the highest performance across all linear axis poses. At four linear axis poses, this complete uncoupled configuration yields a mean IK reachability of 51.8% and data-complete trajectory coverage at 66.3% of evaluated ROI positions.

**Table 1:** Comparison of reachability configurations across cumulative linear axis positions showing mean IK reachability and percentage of ROI positions enabling data-complete trajectories. Mean coverage describes the percentage of viewing directions from the basic set of potential views that are reachable, averaged over all evaluated ROI positions on the car body. Data-complete trajectory percentage represents the proportion of evaluated ROI positions where optimized trajectories exceeding 220$^\circ$ arc length are achievable.

Linear Axis Poses	Configuration	Mean Coverage (%)	Data Complete Trajectory (%)
One pose (2.0 m)	Coupled Rotation and Coupled Orbit	9.1	11.0
	Uncoupled Rotation and Coupled Orbit	10.4	13.6
	Coupled Rotation and Uncoupled Orbit	11.8	16.1
	Uncoupled Rotation and Uncoupled Orbit	13.2	17.2
Two poses (2.0, 3.25 m)	Coupled Rotation and Coupled Orbit	15.4	16.8
	Uncoupled Rotation and Coupled Orbit	19.0	20.1
	Coupled Rotation and Uncoupled Orbit	19.1	23.1
	Uncoupled Rotation and Uncoupled Orbit	22.9	28.2
Three poses (2.0, 3.25, 4.5 m)	Coupled Rotation and Coupled Orbit	24.3	20.1
	Uncoupled Rotation and Coupled Orbit	30.2	26.4
	Coupled Rotation and Uncoupled Orbit	29.2	30.0
	Uncoupled Rotation and Uncoupled Orbit	34.9	36.6
Four poses (2.0, 3.25, 4.5, 5.75 m)	Coupled Rotation and Coupled Orbit	37.8	43.6
	Uncoupled Rotation and Coupled Orbit	44.6	50.9
	Coupled Rotation and Uncoupled Orbit	45.9	59.7
	Uncoupled Rotation and Uncoupled Orbit	51.8	66.3

The performance differences between configurations increase progressively as additional linear axis positions are introduced, see Table 1. At one linear axis position, the difference between the coupled baseline configuration and the uncoupled configuration is 4.1 percentage points for mean coverage, increasing to 14.0 percentage points at four linear axis positions. Similarly, for data-complete trajectory coverage, the gap grows from 6.2 percentage points to 22.7 percentage points.

## Discussion

This work addresses the challenge of performing region-of-interest computed tomography scans on large objects such as car bodies using a twin-robotic CT system, where current investigations often neglect collision-free reachability. Our approach overcomes these limitations by systematically maximizing collision-free reachability for the twin-robotic CT system.

The core contribution of this study is the development of a simulation-based methodology that explicitly decouples reachability determination from trajectory optimization. By first establishing the collision-free workspace through comprehensive inverse kinematics reachability analysis relying on the CAD data of the scan object, subsequent trajectory optimization can operate exclusively within practically achievable configurations. This represents a significant departure from prior approaches that often assume idealized, collision-free geometries. The modular nature of the methodology allows for integration of any trajectory optimization criterion. Here, finding a circular trajectory with maximum data completeness based on the Tuy-Smith condition served as a proof-of-concept, but the framework can be adapted for alternative optimization criteria.

A limitation of this work is that trajectory feasibility is evaluated based on IK-reachability rather than explicit verification of trajectory-reachability. As a result, the reported trajectory lengths represent an upper bound on trajectory-reachability. In cluttered environments, it is possible that individual poses are IK-reachable while no collision-free motion exists between them. However, the proposed methodology aims to generate densely connected regions of IK-reachable poses, which increases the likelihood that trajectory-reachability can be achieved in practice^39–41^. Furthermore, the presence of multiple IK solutions for each pose provides additional flexibility for motion planning, enabling the selection of joint configurations that facilitate feasible transitions between neighboring poses. Explicit verification of trajectory-reachability would require motion planning between pose pairs, introducing computational complexity on the order of $\mathcal {O}(n^2)$ and significantly increasing total computation time for the large set of evaluated poses. Nevertheless, explicit motion planning and verification of trajectory-reachability remain necessary for physical implementation.

The simulation results in this study are specific to the BMW twin-robotic CT system at the Forschungs- und Innovationszentrum^3^ and where evaluated on a BMW 4-series body-in-white. A batch simulation was established, where for symmetry reasons only the right side of the car was evaluated. Here, 273 equally spaced ROI poses were sampled across the car body with a distance of 20 cm between consecutive poses. This allows analysis of the reachability of the complete car body assuming that the volume size of each reconstructed ROI can be spanned between 10–20 cm. The comprehensive validation across these 273 region-of-interest positions and multiple configurations quantifies the benefits of this approach.

By exploiting rotational degrees of freedom (rotation around principal axes) and translational degrees of freedom (variable orbital radii), the methodology achieves substantial improvements over baseline configurations. Quantitatively, the uncoupled rotation and uncoupled orbit configuration at four linear axis positions yields 51.8% mean workspace coverage and 66.3% data-complete trajectory coverage (Table 1), compared to 37.8% and 43.6% for the coupled baseline. Improvements are most prominent in geometrically constrained regions such as vehicle interiors and floor areas, as demonstrated in Figure 12.

The observed improvements can be understood by examining how different degrees of freedom contribute to workspace expansion. Figure 7a demonstrates that rotational degrees of freedom densify the interior of already-accessible regions by filling gaps between viewing directions. This effect is particularly pronounced in difficult-to-access regions with high geometric constraints, where small angular adjustments enable collision avoidance. In contrast, Figure 7b shows that translational degrees of freedom primarily expand region boundaries by enabling access to new viewing positions at different orbital radii. This boundary expansion becomes more effective as geometric constraints relax, enabling longer continuous trajectories.

This complementary behavior is further reflected in the cumulative coverage distributions shown in Figure 10. The crossover between configurations at approximately 36% coverage indicates that uncoupled orbit flexibility is particularly effective for achieving moderate coverage levels at easier-to-access regions. Beyond this threshold, uncoupled rotation flexibility becomes increasingly important for difficult-to-access areas with high geometric constraints. The superior performance of the fully uncoupled configuration across all coverage levels demonstrates that both types of flexibility contribute synergistically. Rotational flexibility enables access to constrained regions, while orbital flexibility extends trajectory lengths once basic access is established, thereby maximizing overall workspace coverage.

We observed that reachability benefits most from the rotational and translational degrees of freedom in difficult-to-access regions such as the interior, where Figure 12 shows that overall coverage is increased for the total uncoupled configuration, especially in the floor region, which positively influenced the maximal optimized trajectory length. This allowed scanning 62 more ROI poses with data-complete trajectories compared to the baseline configuration. However, at the rear of the car body, seven poses remained totally unreachable even after the reachability improvement. This was mainly due to the physical limitations of the twin-robotic CT setup defined here, where the configuration closely follows the real physical setup of the twin-robotic CT system located at the BMW Forschungs- und Innovationszentrum^3^.

The baseline data completeness criterion, where a trajectory is classified as data-complete if it exceeds 220$^\circ$, was introduced as a first-order approximation. While this approach has limitations, for example when the source-detector distance increases such that the active area of the detector might be limited, it provides a solid foundation for trajectory evaluation. This aspect was not considered in this work. Subfigure 10b reveals a steep increase in achievable trajectory arc lengths beyond the 220$^\circ$ threshold, suggesting that many positions may support comprehensive data completeness when the full detector field of view is considered. This indicates that the relatively simple trajectory optimization method employed here provides a strong basis for more advanced optimization strategies.

An important insight from this work is that additional degrees of freedom are only effective when systematically exploited. Table 1 illustrates this by comparing configurations with identical access to rotational, translational, and system-specific degrees of freedom such as linear axis positions. Despite this equivalence, the coupled baseline configuration achieves substantially lower workspace coverage than the uncoupled configuration, demonstrating that mere availability of degrees of freedom does not guarantee improved reachability. Without systematic exploitation, additional flexibility remains underutilized. This effect is also evident in the analysis of the linear axis, where a pronounced increase in reachable trajectories is observed, particularly when extending from three to four axis positions. This increase can be attributed to improved accessibility of geometrically constrained regions, especially at the rear of the car body, where the detector robot would otherwise need to extend over large portions of the structure, leading to increased collision risk. The proposed methodology effectively translates available flexibility into practical workspace expansion, enabling previously unreachable poses.

### Outlook

The methodology presented in this work establishes a foundation for several promising research directions that could further enhance twin-robotic CT systems for large object inspection.

**Integration of Linear Axis into IK Solver.** This work revealed, although unintentionally, the critical importance of the linear axis for workspace expansion. In future work, the linear axis should be integrated directly into the IK solver algorithm, providing each robot with a seventh degree of freedom instead of six. While this would increase computation time for individual IK solving runs, it would reduce the number of separate requests for different linear axis poses and potentially decrease total computational time. Additionally, this would enable continuous sampling of the complete linear axis range rather than the four discrete poses used in this work.

**Additional Degrees of Freedom.** Adjustments to object positioning or inclusion of additional system degrees of freedom, such as lifting tables or turntables, could further expand reachable workspace. The modular nature of the methodology allows straightforward integration of such extensions. Particularly, detector rotations around axes $\vec {n}_x^D$ and $\vec {n}_y^D$ could increase collision-free poses in geometrically constrained regions such as vehicle interiors while simultaneously improving spatial resolution through oblique projection geometries.

**Physical Implementation and Validation.** The results presented in this work are based on a simulation environment that incorporates geometric and kinematic constraints of the twin-robotic CT system. While this enables physically grounded reachability analysis, the methodology has not yet been validated through physical scan execution, and experimental validation on a real system is currently ongoing work. The primary objective for future work should be demonstrating the methodology in a real application on the BMW twin-robotic CT system. The current focus on individual pose reachability rather than continuous trajectory reachability means that explicit motion planning remains essential prior to physical deployment. To transfer simulation results to physical scans, digital twin accuracy is critical. The simulated world and physical world must be closely aligned to avoid collisions, requiring precise referencing of the physical object’s position using measurement tools to achieve millimeter-level accuracy^34,35^.

**Calibration Requirements.** Physical implementation will require geometric calibration to compensate for robot positioning uncertainties. Multiple geometric calibration methods have been established for twin-robotic CT systems^27,29,51^. Furthermore, trajectories exploiting high degrees of freedom may require additional offset and gain calibrations or compensation within the reconstruction algorithm^52^.

**Advanced Trajectory Optimization.** While this work employed data completeness as a proof-of-concept criterion, the framework supports integration of more sophisticated optimization objectives. Future work should apply advanced trajectory optimization techniques such as edge detection for enhanced feature visibility^14^, detectability indices for task-specific quality optimization^19,24^, or other optimization methods^1,8,25^. The reachability maximization approach provides a foundation applicable to any such criterion. Furthermore, the availability of multiple IK solutions per pose, as computed during the reachability analysis, provides additional flexibility for trajectory planning. These solutions could be exploited to select joint configurations that minimize axis movements between consecutive poses, thereby improving trajectory smoothness and as well as scanning time.

**Generalization to Other Objects.** The methodology should be expanded to different large objects where different sets of degrees of freedom might be introduced to increase reachability for task-specific inspection requirements. This would validate the generalizability of the approach and demonstrate its applicability beyond automotive components.

These research directions would progressively close the gap between the theoretical framework presented in this work and a practically ready-to-use method, ultimately enabling demonstration of a fully operational twin-robotic CT system for large object inspection.

## Conclusion

This paper presents a systematic methodology to maximize collision-free reachability in twin-robotic computed tomography systems by exploiting the kinematic degrees of freedom of twin robots together with the X-ray imaging degrees of freedom. The imaging degrees of freedom comprise rotational adjustments (detector rotation around principal axes) and translational adjustments (variable source-detector distances). Implemented fully in simulation and demonstrated on a BMW 4-series body-in-white, the methodology efficiently determines reachable workspaces based on CAD data, enabling optimized region-of-interest scan trajectories

By leveraging rotational flexibility, including independent detector rotations, and translational flexibility through variable source-detector distances, the approach significantly expands the collision-free workspace and increases the number of feasible data-complete trajectories compared to baseline configurations. The modular design decouples reachability analysis from trajectory optimization, supporting integration of various optimization criteria tailored to specific inspection tasks.

Through comprehensive batch simulations across 273 ROI positions and multiple system configurations, we quantify substantial reachability improvements. Data-complete trajectory coverage increases from 43.6% for the baseline configuration (coupled rotation and coupled orbit at one linear axis position) to 66.3% for the fully uncoupled configuration at four linear axis positions (see Table 1). The results demonstrate that systematically exploiting all available degrees of freedom is critical for enabling high-quality CT scans of large industrial components, particularly in challenging geometries such as vehicle interiors. This work advances the connection between theoretical trajectory optimization and practical system constraints by introducing a collision-aware reachability framework based on CAD data and robot kinematics. While full physical validation remains future work, the proposed methodology provides a necessary intermediate step by constraining trajectory optimization to configurations that are feasible under realistic geometric and kinematic conditions. The CAD-based framework generalizes to arbitrary twin-robotic configurations and scan objects, providing a foundation for practical deployment. Future work will focus on experimental validation and extension to additional degrees of freedom, advancing towards fully automated industrial CT inspection.

## Data Availability

The datasets used or analyzed here may be requested from the corresponding author upon reasonable request, subject to the applicable terms of use for BMW Group information content.
